# Caloric Restriction Mimetics Attenuate the Hallmarks of Aging

**DOI:** 10.1093/geroni/igab046.1974

**Published:** 2021-12-17

**Authors:** Guido Kroemer

**Affiliations:** University of Paris, Villejuif/Paris, Ile-de-France, France

## Abstract

Nutrient depletion, which is one of the physiological triggers of autophagy, results in the depletion of intracellular acetyl coenzyme A (AcCoA) coupled to the deacetylation of cellular proteins. We found that there are at least 4 possibilities to mimic these effects, namely (i) the depletion of cytosolic AcCoA by interfering with its biosynthesis, (ii) the stimulation cytosolic AcCoA consumption, (iii) the inhibition of protein acetyltransferases, or (iii) the stimulation of protein deacetylases. Thus, AcCoA depleting agents, AcCoA-consuming agents, acetyltransferase inhibitors or deacetylase activators are highly efficient inducers of autophagy and reduce aging-associated diseases including diabetes, obesity, cardiac failure and failing cancer immunosurveillance. Hence, we classify them as "caloric restriction mimetics" (CRM). We have initiated the systematic search for CRMs based on their cellular effects in vitro. We built screening assays amenable to high-throughput technology for the identification of CRMs. These results will be discussed.

